# Advancements in Wound Dressing Materials: Highlighting Recent Progress in Hydrogels, Foams, and Antimicrobial Dressings

**DOI:** 10.3390/gels11020123

**Published:** 2025-02-07

**Authors:** Adina Alberts, Dana-Ionela Tudorache, Adelina-Gabriela Niculescu, Alexandru Mihai Grumezescu

**Affiliations:** 1 Carol Davila University of Medicine and Pharmacy, 050474 Bucharest, Romania; adina-magdalena.alberts@rez.umfcd.ro; 2 National University of Science and Technology Politehnica Bucharest, 011061 Bucharest, Romania; dana.tudorache@upb.ro (D.-I.T.); agrumezescu@upb.ro (A.M.G.); 3 Research Institute of the University of Bucharest—ICUB, University of Bucharest, 050657 Bucharest, Romania

**Keywords:** wound healing, wound dressings, hydrogels, foam dressings, antimicrobial dressings, stimuli-responsive materials, biofilm management, drug delivery systems, nanotechnology in wound care

## Abstract

Recent advancements in wound dressing materials have significantly improved acute and chronic wound management by addressing challenges such as infection control, moisture balance, and enhanced healing. Important progress has been made, especially with hydrogels, foams, and antimicrobial materials for creating optimized dressings. Hydrogels are known for maintaining optimal moisture levels, while foam dressings are excellent exudate absorbents. Meanwhile, antimicrobial dressing incorporates various antimicrobial agents to reduce infection risks. These dressing options reduce wound healing time while focusing on customized patient needs. Therefore, this review highlights the newest research materials and prototypes for wound healing applications, emphasizing their particular benefits and clinical importance. Innovations such as stimuli-responsive hydrogels and hybrid bioengineered composites are discussed in relation to their enhanced properties, including responsiveness to pH, temperature, glucose, or enzymes and drug delivery precision. Moreover, ongoing clinical trials have been included, demonstrating the potential of emerging solutions to be soon translated from the laboratory to clinical settings. By discussing interdisciplinary approaches that integrate advanced materials, nanotechnology, and biological insights, this work provides a contemporary framework for patient-centric, efficient wound care strategies.

## 1. Introduction

A wound can be described as a disturbance of skin structure, an injury that leads to loss of skin continuity, mucous membrane, or tissue. This can be caused by external factors, such as thermal, chemical, physical, electrical, thermal, pressure, or other medical factors or physiological disorders (malignancies, diabetes, etc.). Wounds can be classified into two categories based on healing time, namely chronic and acute wounds [1,2,3,4]. Acute wounds have a healing time between 2 and 3 weeks for normal healthy people, and if the time is longer than 6–8 weeks and has an abnormal healing process, it can be defined as a chronic wound or non-healing wound [2,5].

The process of wound healing is divided into four main stages. Each step involves different cell types, namely hemostasis (clot formation), inflammation, proliferation (new tissue formation), and remodeling (skin repair) [6,7,8,9]. Unfortunately, there are several cases where an acute wound can become chronic because of several pathological factors, such as infection, metabolic disease, vascular insufficiency, nutrition or neurological deficiencies, age, etc. Also, there are other factors, the external ones like humidity and temperature, that can transform into infected wounds, burn wounds, diabetic wounds, pressure ulcers, and arterial/venous ulcers, resulting in pain, exudate production, necrosis, amputation, or even death [1,3,5,10,11].

Regarding all the external and pathological factors, achieving a cost-effective, reproducible, non-invasive, clinical, and approved treatment for severe wounds, e.g., diabetic ulcers and chronic and even severe burns, remains a challenge despite all the significant advancements of researchers. Over time, various materials and methods have been largely researched to prevent infections and scarless wound healing. According to existing research, hydrogel dressing has proven many required properties for applications in wound dressing materials, such as adsorbing wound exudate capacity, the healing process, contributing to gas exchange, maintaining a moist surface, biocompatible, biodegradable and even avoiding microorganism contamination [12,13].

Wound dressing can be characterized as a therapeutic tool with a role in adsorption, drainage, and protection, and it facilitates the healing of wounds. Regarding all the attributes, the main important characteristics that wound dressing should respect are biocompatibility, similar biochemical and microstructure properties to support the synthesis of extracellular matrix (ECM), motility, and cell proliferation, absorb exudate, non-allergic, non-toxic, sterile, is necessary to protect the local injury from microorganisms, to maintain the wound moist. Also, it is important to remove the dressing from the injured site without causing a second wound harm [14,15,16].

Throughout history, wound healing has posed significant challenges. Various materials have been utilized to obtain good wound dressing, from natural ones that can be easily found in ancient times to advanced materials that are synthesized in specific and authorized working places. For example, the ancient Egyptians utilized natural materials such as milk, grease, honey, herbs, mud, lint, or clay for dressing. The ancient Greeks used vinegar and wine for wound washing. It is known that the sugar complex from honey suppresses the growth of bacteria by applying osmotic pressure, causing water flow out of bacteria cells. Resveratrol, a natural polyphenolic antioxidant found in red wine, is known for inhibiting *Pseudomonas aeruginosa*, *S. aureus*, and *Enterococcus faecalis*. In Indian literature, chili powder is utilized in dog bites or other rabid animals as dressing material, and turmeric powder is used for its hemostatic and antiseptic properties [14,17,18,19,20,21,22].

Louis Pasteur identified the necessity of sterilization and pasteurization in surgical practices, and Joseph Lister became so fascinated by Pasteur’s work that he proposed heat, filtration, or even chemicals to destroy or eliminate the airborne in such a way that an antiseptic barrier between the wound and the incision surrounding air. Regarding this, he proposed sterilizing tools and clothing by heat, applying phenic acid as a spray and impregnant dressing, wrapping after with a thin tin foil, and establishing washing and wound cleaning as a routine action. Both of them have an essential role in history because they set up a scientific base for the management of wounds [18,23,24].

In 1962, George Winter introduced the idea of moist healing, which challenged the conventional notion of dry wound healing. Various categories of moist dressing, considered modern dressing, can be included, such as hydrogels, foams, films, hydrocolloids, alginates, and nanofiber. Moreover, moist dressing promotes healing, inhibits microbial activity, and assists the progress of wound closure [4,17,25].

However, simple moisturizing dressings cannot inhibit the growth process of bacteria, and under those conditions, the wound can lead to chronic or non-healing without an effective treatment strategy. An essential role in expanding and supporting the entire healing process of wounds is the pharmacological agents, such as anti-inflammatory and antibacterial agents, and even various biological agents, such as growth factors, that accelerate damaged tissue regeneration. Nowadays, the incorporation of microelectronics has led to the appearance of a new class of wound dressing, smart wound dressing, which can monitor the indicators at the wound location and administer therapeutics agents to facilitate the wound healing process [26,27,28].

Wound dressings, including foams, hydrogels, and antimicrobial dressing, represent significant progress in wound healing, offering enhanced healing strategies and superior patient results. Hydrogels are known for maintaining moisture levels, while foam dressings offer superior exudate absorption. On the other hand, antimicrobial dressing incorporates various antimicrobial agents to reduce the risk of infection. These advanced dressing types facilitate wound healing and focus on patient needs, such as infection control.

The development of smart wound dressings represents a significant innovation, as they have the potential to monitor wound conditions in real-time and deliver target therapeutic agents to accelerate healing. Thus, this paper aims to contribute to the field by overviewing the most recent findings, including the incorporation of therapeutic agents, such as antimicrobial agents and growth factors, and microelectronics into wound dressings, which could transform the management of chronic wounds by providing personalized care. This literature investigation aims to stand as an inception point for further studies toward developing advanced wound dressings that combine hydrogel materials, biologically active agents, and smart technologies. Granting State-of-the-Art wound management alternatives, in-depth future research studies can build upon existing knowledge to create affordable, reproducible, clinically approved treatments for chronic and severe wounds, enhancing infection control, tissue regeneration, and personalized care through real-time monitoring and therapeutic agent delivery. Therefore, this review highlights the newest research materials and prototypes to offer and updated framework in the field of advanced materials that can be used daily by patients in need.

## 2. Key Innovations in Hydrogels

Hydrogels can be defined as 3D networks composed of chains of hydrophilic polymers that quickly absorb a large quantity when they come in contact with water, going into a partly solid material. Due to their three-dimensional structure, biocompatibility, good permeability, shape adaptability, mechanical protection, moist environmental ability, and air permeability, hydrogels are considered excellent dressing candidates for wound dressing [16,29,30,31]. Because of their characteristics, hydrogels started to gain increasing interest in the wound dressing field, and the section below highlights the necessity of drug-loaded hydrogels, the development of hybrid and stimuli-response hydrogels, their application, and their challenges.

### 2.1. Recent Materials

**Figure 1:**
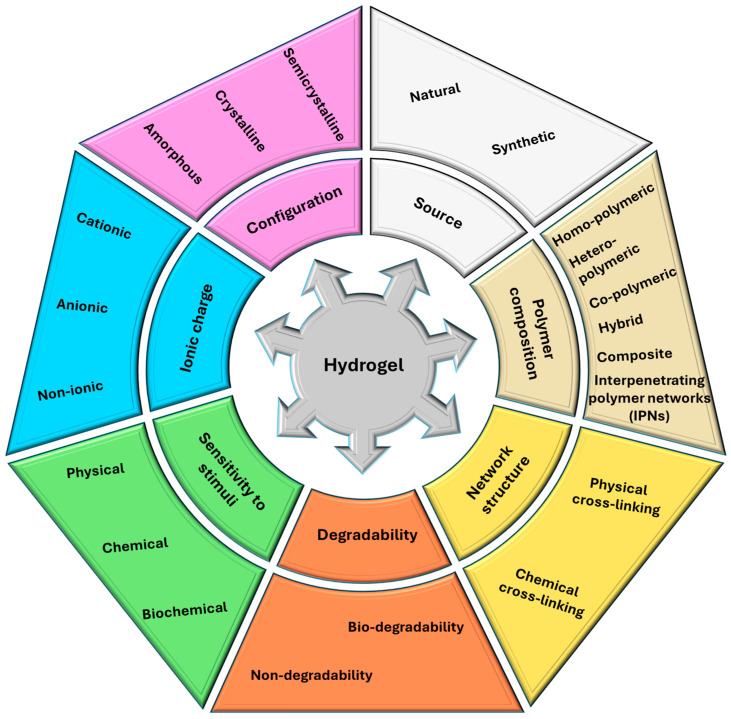
Schematic representation of the classification of hydrogels. Created based on information from [32,33,34].

Hydrogels can be divided into subcategories, classified on criteria like source, the composition of polymers, network structure, configuration, ionic charge, degradability, sensitivity to stimuli, and physical aspect [32,33], as depicted in Figure 1.

Natural hydrogels present a wide range of characteristics, such as biocompatibility, hemostasis, anti-inflammation, high water content, semi-transparency, and transparency, that help monitor easily the wound healing microstructure, similarly to the ECM and a variety of natural polymers that can be used, such as collagen, chitosan, fibrin, gelatin, cellulose, and hyaluronic acid. There are also some challenges regarding practical applications, such as antibacterial capacity improvement for preventing wound infection, increased biodegradability to assist the progress of natural degradation after use, and improved mechanical properties, as they can be utilized for different wound types [35,36,37,38].

Contrary to natural polymers, synthetic polymers can be easily synthesized at a large scale by different processes, cross-linking, functionalization, or polymerization, and their physicochemical properties and stability can be tailored in a controlled manner. Also, their reproducibility represents a good advantage regarding their application in the medical field. Also, synthetic polymers can create an impermeable wall to water and bacteria, in addition to enabling sufficient aeration and the transport of healing agents. Unfortunately, synthetic polymers also have drawbacks, such as the absence of bioactivity, compared with natural polymers [32,39,40].

With all the advances and challenges that natural and synthetic polymers present, combining them results in a new hybrid class of hydrogels. These combinations lead to increased mechanical abilities, improved flexibility, biocompatibility, biodegradability, quicker wound healing, and high adsorption capacity, and they also promote healing [41,42,43].

Moreover, smart hydrogels have been developed as the most important subdivision of hydrogels, particularly for development in robotic and biological applications, because they can be used as a powerful tool that reduces the limitations of conventional wound treatment. Stimuli-responsive hydrogels, as their name suggests, are smart materials that can change their mechanical properties, swelling capacity, and hydrophilicity when exposed to various stimuli like pH, temperature, light, enzyme, antigens, magnetic, electric, redox, ultrasound, and multi-responsive materials [44,45,46,47,48].

### 2.2. Drug Loaded Hydrogels

Integrating antimicrobial agents and growth factors into hydrogel wound dressings significantly improves their therapeutic effectiveness, facilitating more efficient wound healing while concurrently reducing the risk of infections. While antimicrobial agents primarily focus on treating systematic or local infections, growth factors enhance cellular regeneration and accelerate tissue regeneration. This combination boosts the dressing’s ability to combat microbial growth and supports the regenerative processes essential for wound healing [26,49,50].

Antimicrobial agents primarily focus on treating systematic or local infections, problems that might be associated with delay in healing time, swelling, pain, or increasing pain. On the other hand, the growth factors enhance cellular regeneration and accelerate tissue regeneration, and they can also help avoid extra infection [51,52,53,54].

#### 2.2.1. Antimicrobial Agents

From the first time of skin injury, the place starts to be inhabited by bacteria from the surrounding skin, the air, or other materials that are not property or not at all sanitized. Also, an inflammatory reaction is imperative for adequate wound healing, but excessive inflammation can delay wound healing. Many inflammatory factors critically affect the wound repair process, including reactive oxygen species (ROS), which are liberated in wound inflammation. But, sometimes, the wound can start to be infected and require drugs or other antibacterial reagents, such as nanoparticles [55,56,57].

A few metallic nanomaterials, like silver, gold, copper oxide, or zinc oxide, are known for their antimicrobial and biological properties that can be used as drug-loaded hydrogels regarding their applicability in the reduction in the infection and accelerating the process of wound healing. Silver nanoparticles are acknowledged for their chemical stability, low cost, and especially for their great antibacterial activity, both in vitro and in vivo, against a broad spectrum of bacteria, such as *Staphylococcus aureus*, *Escherichia coli*, *Salmonella typhus*, *Pseudomonas aeruginosa*, *Vibrio cholera*, etc. [58,59,60,61,62,63,64,65,66].

Besides silver nanoparticles, as briefly mentioned above, other nanoparticles show antimicrobial activity for several Gram-negative and Gram-positive bacteria [51]. For example, copper nanoparticles can easily pass through the cell membrane if bacteria and viruses are present, effectively exterminating them by producing oxygen-releasing hazardous chemicals [67,68,69]. Zinc oxide can produce cell death by generating ROS, which damages the cell membrane [70,71,72]. Also, by effectively killing relevant pathogens, hydrogels containing gold nanoparticles stop bacteria from growing [51,73,74].

Another strategy that can be used to prevent wound infection is antibiotic-loaded hydrogels, which have shown real success in antibacterial therapy since Alexander Fleming discovered ampicillin. Antibiotics represent a powerful tool regarding wound healing infection, and the antibiotics are segmented into five categories, namely beta-lactams, tetracyclines, fluoroquinolones, macrolides, and aminoglycosides. The most widely used include Gentamicin, Ciprofloxacin, Vancomycin, and Moxifloxacin [75,76,77].

Antibiotics are generally administrated in two ways, injectable or direct oral, for an effective treatment. Unfortunately, the direct utilization of antibiotics makes it difficult to control drug usage, which can lead to bacterial resistance. Therefore, incorporating antibiotic medications into the hydrogel’s porous structure represents a better administration alternative. This allows for direct administration of the medication at the site of illness, which minimizes the rate of overuse of antibiotics and improves antibiotic utilization while maintaining good biocompatibility. Also, drug-loaded hydrogels facilitate regular and controlled drug molecule administration into wound sites for a long time without requiring dressing material replacement [76,78,79].

#### 2.2.2. Growth Factors

Another promising therapeutic approach for wound healing is the inclusion of growth factors. The growth factors are vital in every phase of wound healing, by proliferation, conducting the system of mesenchymal cells, and the production of ECM. Hydrogels can be loaded with therapeutics drugs, cells, different molecules, and growth factors that accelerate the closure of the wound and avoid infection. Platelet-derived growth factor (PDGF), transforming growth factor-beta 1 (TGF-β1), vascular endothelial growth factor (VEGF), basic fibroblast growth factor (bFGF), and epidermal growth factor (EGF) demonstrated essential role in the process of regeneration [53,80,81,82].

Platelets are delivered at the injury and stick to the blood vessel walls that have been damaged because they play a crucial part in primary coagulation. To promote tissue regeneration, the clot formed during the coagulation phase first secretes several kinds of cytokines and growth factors, including PDGF and EGF. Epidermal growth factors facilitate the renewal of epidermal cells by stimulating the keratinocyte migration and proliferation while stimulating the granulation tissue formation and fibroblast cell motility. FGF promotes fibroblast proliferation and migration at the wound place once it is released from the hydrogel. The transforming GFs bring immune cells from the blood vessels and the nearby tissue and initiate the inflammatory stages of healing [53,79,80,83,84,85]. Thus, incorporating various relevant growth factors into the composition of hydrogels can endow these materials with supplementary valuable properties to aid the wound healing process.

**Figure 2:**
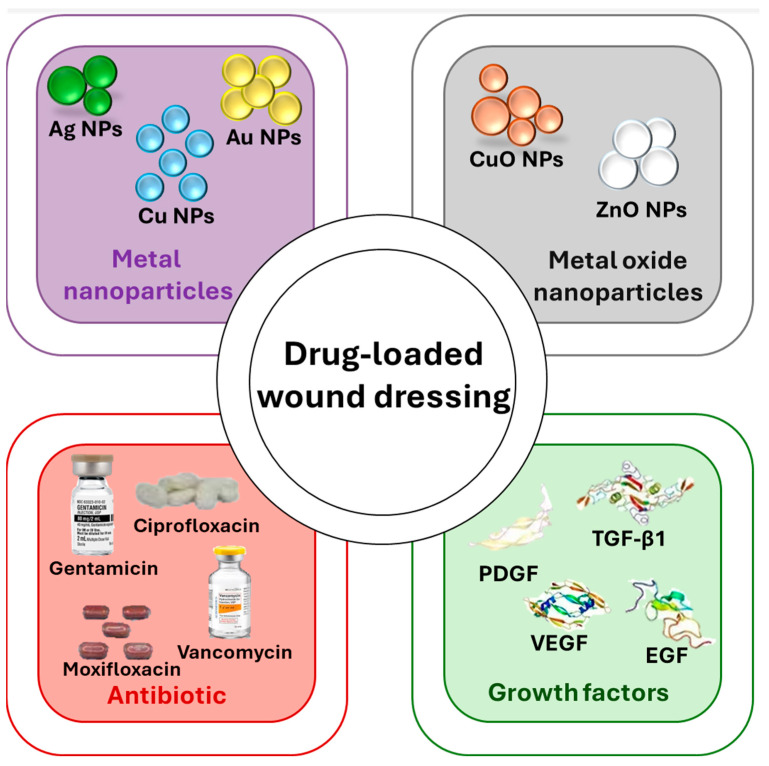
Schematic overview of various types of drug-loaded hydrogel dressings. Created based on information from [26,76,86].

Figure 2 presents a schematic overview of the various drug-loading possibilities for hydrogel materials, visually synthesizing the key findings for an at-glance perspective.

### 2.3. Applications in Recent Studies

Chronic wounds, such as diabetic foot ulcers and diabetic complications, typically exacerbate because of ischemia, inflammation near the wound, nitric oxide blockage, DNA modification, ROS, and an upregulation of kinase C. This type of wound can have a significant and negative impact on the quality of life of patients, and it can cause social care costs to increase. Also, the presence of bacteria can lead to considerable injury [87,88,89].

Several interventional studies have been performed on diabetic foot ulcers using different drugs, hydrogels, drug-loaded hydrogels, and even hydrogel-based nanoparticles. Table 1 summarizes clinical trial information, including the number of participants, the date when the study was finished, the treatment or intervention applied, and the study phase.

As described in Table 1, nine studies have been identified, out of which only three are in Phase 4. These are expected to bring products to the market soon, while the rest of the investigated solutions still require more advanced studies before entering the clinical setting. Also, clinical trials with less than 50 enrollments need a larger number of participants for relevant statistical conclusions. All these results are essential and represent an important step in the technological transfer of materials from the laboratory to their use by humans.

Burns is recognized as one of the most prevalent causes of trauma on a global scale, impacting millions of individuals each year. Burn wounds can develop from acute to chronic illnesses and can become infected if there are microbial and growth invasions. The cooling and moisture properties of hydrogel can effectively ease pain and also promote wound healing [90,91,92,93]. Table 2 below presents various clinical trials performed on burns regarding wound healing and hydrogel’s cooling or moisturizing properties.

Table 2 above shows that clinical trials have been conducted in different countries from three of seven continents, namely Asia, Europe, and North America, which highlights the importance of geographic diversity in enhancing the generalization and applicability of healthcare findings. Another important aspect that can be easily observed is the number of enrolments, which, in most cases, is below or approximately 20 participants. This small number of participants offers insights only into certain cases, requiring more in-depth investigations and larger enrollments to ensure the relevance of pertained conclusions for broader populations.

Three of the ten studies were conducted in Seoul, South Korea, representing a continuous observation of an ALLO-ASC-DFU prototype. These clinical trials started in 2015, and after almost 8 years, they are still in phase one, marking a stale in the product advancement.

Furthermore, beyond the extensive clinical trials examining the clinical utility of hydrogels, several hydrogel-based wound dressing products have already been introduced to the market, as exemplified in Table 3.

### 2.4. Challenges Addressed

Mechanical stability and degradation profiles represent two of the main challenges that hydrogel wound dressing presents and are encountered often. Ideally, wound dressing should gradually yield space for newly formed ECM and proliferating cells. Combining natural and synthetic polymer properties makes hybrid hydrogels more robust and flexible, and they have good biodegradability, biocompatibility, and a high adsorption capacity. Also, including a secondary phase, such as drug-loaded nanoparticles or biological molecules, within the hydrogel matrix, the resulting material displays excellent skin regeneration and wound healing [43,102,103].

The change in form or mechanical properties of hydrogels is often accomplished by chemical and/or physical processes, each of them offering its advantages in terms of final material properties and performance. Chemical cross-linking denotes the intramolecular or intermolecular bonding of two or more molecules, and the hydrogels obtained via this method present high mechanical stability and remarkable mechanical strength. In contrast to chemical crosslinking methods, physical crosslinking offers greater ease of recovery and reconstruction regarding mechanical properties. This approach is frequently used to fabricate mechanically elastic hydrogels [104,105].

Another approach involves the incorporation of textile reinforcement to enhance the mechanical properties of hydrogels. The strategy leverages the inherent strength and elongation, which does not easily fall out and will not induce a secondary injury. Natural fibers, such as cotton, flax, hemp, and jute-based, are environmentally friendly, low-cost, biodegradable, and low-density, making them good candidates for composites [104,106,107,108]. For example, Yuan et al. [107] have obtained cotton fabric-reinforced hydrogels that demonstrate both photothermal antibacterial and excellent mechanical properties. Also, Ahmad et al. [109] produced a titanium oxide-loaded cellulose hydrogel reinforced with nonwoven cotton with absorptive capability, moisture management, and mechanical strength, which have increased.

Besides the flexibility of hydrogels, as mentioned below, the degradation profile represents another challenge that needs to be resolved to obtain a non-toxic hydrogel. For instance, Chen et al. [110] obtained an antibacterial polyvinyl alcohol/chitosan hybrid hydrogel incorporated with different nanocomposites, iron, copper, and zinc oxide. Their study showed that a ratio of 4:1 (PVA:CS) with 0.3% solid content demonstrated non-cytotoxic and excellent properties. Also, the antibacterial agents exhibit good antimicrobial properties against *S. aureus* and *E. coli*.

Differently, Mushtaq et al. [111] developed an injectable chitosan–methoxy polyethylene glycol (chitosan–mPEG) hybrid hydrogel with tailored physicochemical and mechanical properties, compared with solo PEG or chitosan hydrogel.

An interesting alternative was also proposed by Rusu et al. [112]. The researchers developed a well-defined maleoyl-chitosan/poly(aspartic acid) nanogel integrated into a thiolated hyaluronic acid (HASH)-based polymer network with amoxicillin-controlled drug release. This new material exhibits a controlled degradation rate and reduced swelling capacity compared with non-filled HASH.

## 3. Progress in Foam Dressings

In wound dressing, foams are formed in two phases: from a solid polymer and a dispersed air. These materials are used specifically for moderate and minimal wounds, such as chronic wounds, burns, or even deep ulcers, and they are not good for necrotic wounds, epithelial dry wounds, or even for frequent requiring care [2,113,114].

**Figure 3:**
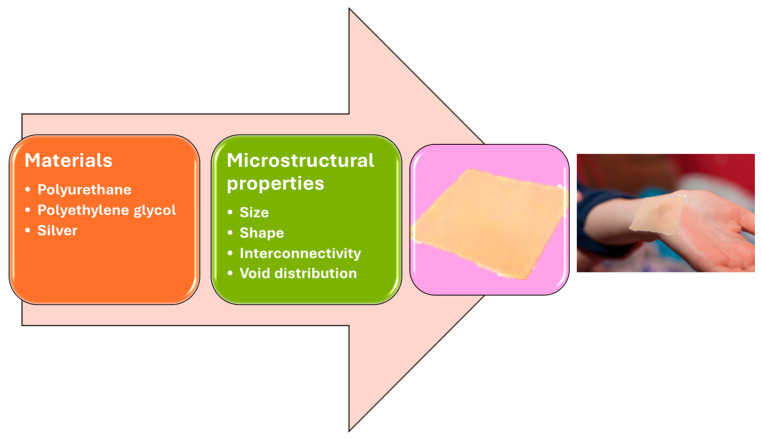
Examples of parameters that determine the final function of foam wound dressings. Created based on information from [119,120,121].

Regarding the application of wound foam and the fact that the primarily indicated utilization is for exudate management, there are essential parameters that determine the final function. Their microstructural aspects, for example, shape, size, interconnectivity, and void distribution, are essential for an optimal dressing concerning the application, such as stiffness, compressibility, and permeability, directly determined by the void’s connectivity, density, and arrangement [113,115,116,117,118]. Figure 3 illustrates the important aspects of obtaining a well-performing foam wound dressing.

### 3.1. Material Advances

One of the most used polymers for foam manufacturing is polyurethane (PU). This polymer provides several characteristics that are essential for wound care and healing. Biocompatibility, flexibility, softness, water absorptivity, gas permeability, physiochemical properties, and economic advantage are also important [115,122,123].

Foam/sponge-based exudate dressing presents a high capacity for fluid absorption, and their 3D structure makes them ideal candidates in deep wounds and considerable exudate volumes. Studies have shown that using natural polyols like alginate and hydroxypropyl methylcellulose in foam production improves the foam dressings’ absorption capacity and pressure resistance. Polyethylene glycol was also studied for its moisture absorption capacity when in contact with body fluids or blood, which expands volume compared to its original size [122,124].

Besides the tailored absorption capacity, there are some cases where the infection represents a real problem in wound healing. By incorporating antimicrobial agents directly into the polyurethane structure, such as different chemicals (polyhexamethylene guanidine compounds, quaternary ammonium, and metal nanoparticles) or even natural products (e.g., plant extract, biopolymers), antimicrobial properties can be induced. Different metallic nanoparticles, such as silver, zinc oxide, gold, copper oxide, and titanium dioxide, have been studied for their antimicrobial activity in polyurethane foams and also their improvement in mechanical strength and structural properties. The most frequently used is silver due to its broad-spectrum antibiotic activity [125,126,127].

### 3.2. Innovative Applications

Concerning the primary application of foam dressings, Table 4 below provides a detailed overview of several clinical trials that specifically investigate their effectiveness in treating venous leg ulcers and infected pressure ulcers.

The diversity in enrollment numbers suggests differing levels of participant engagement and potentially varying degrees of clinical relevance. Studies with larger enrollment may indicate greater generalizability and robustness of the findings, whereas smaller trials may serve more specific hypotheses or niche applications. Also, geographical diversity illustrates a diverse clinical landscape and applicability to various healthcare settings, thereby supporting the broader adoption of effective treatments.

Regarding foam dressing that enhances cellular migration and reduces inflammation, it was observed that hydrocellular foam dressing has been utilized in the management of lesions that exude moderate to heavy fluid. Also, these hydrocellular foam dressings can reduce inflammation by reducing the gene expression level of inflammatory markers, interleukin [128,129]. Table 5 above demonstrates a few clinical trials of hydrocellular foam that have been used in different applications of chronic wounds.

The results presented in Table 5 show that hydrocellular foam dressing may be a promising chronic wound dressing based on significant enrollment sizes with a number above 40 participants. Also, completion dates from 2016 to 2021 indicated a recent and ongoing interest in optimizing wound dressing. Also, the diversity in locations accentuates the global applicability of these findings, demonstrating the potential applications for these dressings.

Table 6 illustrates several foam dressings that are available on the market at present.

### 3.3. Enhanced Properties

The stiffness and strength of the final foam are determined by the raw material and by the manufacturing processes, which can affect the microarchitectural properties of the foam porosity and even the solid polymer phase properties. The microarchitecture characteristics are very important because they determine the collected and stored volume of the exudate. For example, if the foam is denser, the foam has less air and increases stiffness. Also, regarding the external forces, such as external mechanical forces that appear when the foam is compressed for the final form, the microarchitecture spaces can collapse or diminished, and the absorbance capacity is further reduced [113,115].

Regarding the improvement of the function, some modifications have been made. For example, for a strength increase in adherence to wound dressing, a sticky, adherent silicon rim is created along the foam dressing’s edges [115]. Also, by adding silver nanowire, Chen et al. [136] obtained a foam dressing that demonstrates excellent bending-compression durability, long-term stability in wet environments, good biocompatibility, and exceptional electrical qualities, which make it appropriate for wound healing.

## 4. Cutting-Edge Antimicrobial Dressings

### 4.1. Recent Innovations

Nanotechnology enhances rapidly advanced research, which is applied both in diagnostic and therapeutic applications. Nanotechnology enables the production and use of nanoparticles, smart dressing, and biomolecule-loaded dressing to accelerate the wound healing process [137,138,139]. Furthermore, it is essential that drug release is well-controlled and that the dose is properly administered during the repair process. Smart dressings are three-dimensional and can change their mechanical stability and size depending on the environmental condition by monitoring biomarkers (e.g., glucose, pH, oxygen, inflammation factors, toxins, uric acid, enzymes secreted by bacteria) and physiological conditions (moisture and temperature). An essential property of this smart wound dressing is the controllable delivery of antimicrobial drugs based on infection monitoring [46,79,140,141].

#### 4.1.1. pH-Responsive

pH is an important factor regarding the correct skin function, and it can vary despite the skin layers. Also, a variation in pH appears in different wound healing phases. For example, healthy skin has a pH value of 5–6, acute wounds around 7.4, while chronic has an alkaline pH of 7.3–10 when the bacteria colonies proliferate. Regarding this, pH-stimuli response wound dressing will deliver an antimicrobial drug when the pH increases [44,142,143].

#### 4.1.2. Temperature-Responsive

Temperature represents a vital factor in the wound healing phases because it influences enzymatic and chemical processes, as well as the cellular activity related to healing, making temperature monitoring essential for understanding oxygenation, local blood flow, inflammation, and infection. For example, the temperature of a wound without infection is almost the same as that of normal skin, 37.8 °C. An increase of 2.2 °C indicates that the wound has an infection or an inflammatory response [144,145,146].

#### 4.1.3. Glucose-Responsive

Hyperglycemia is characteristic of diabetes patients, leading to the utilization of glucose-responsive hydrogel in the treatment of diabetic ulcers. Because glucose contains an ortho–diol group, it allows for more hydrophilic structure formation when interacting with phenylboronic acid following ionization and promoting drug release [147].

#### 4.1.4. Enzyme-Responsive

**Figure 4:**
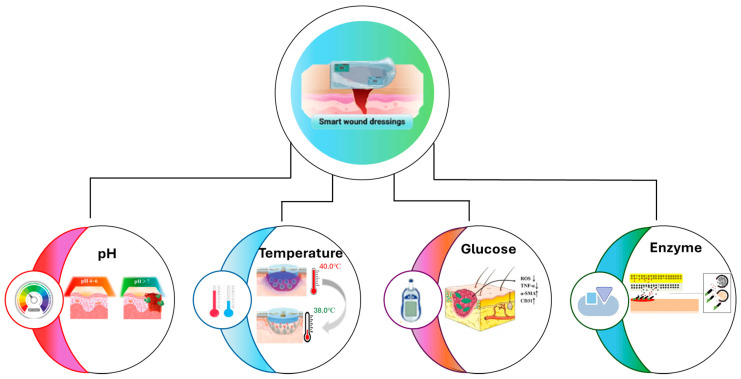
Visual representation of smart wound dressings. Created based on information provided from [44,104,144,149,150].

Enzyme-responsive hydrogels undergo degradation exclusively in the presence of specific enzymes while maintaining stability in alternative environmental conditions. The expression levels of these enzymes vary based on the severity of the wound, which in turn influences the hydrogel’s capacity for drug release, thereby facilitating controlled release mechanisms. Regarding this, enzyme-responsive hydrogels present an advantage because they can be designed to degrade specific enzymes, permitting therapeutic release [147,148]. Table 7 and Figure 4 below illustrate various smart wound dressings that release antimicrobial agents for different stimuli responses.

**Table 7:** Examples of smart antimicrobial systems that release agents only in the presence of infection.

Smart Wound Dressing	Stimuli Response	Active Components	Targets Wound Type	Benefit	References
3D printed alginate wound dressings doped with calcium phosphate nanoparticles (CaP NPs)	pH-responsive	selenium nanoparticles, Rhodamine B, Bacitracin	Under basic condition	3D printability is adaptable to various sizes and shapes	[151]
Cellulose-functionalized-Graphene oxide	pH-responsive	Curcumin	Chronic wounds, burn	The curcumin was realized in a controllable manner	[152]
Zwitterionic chitosan and dialdehyde starch with silymarin and levofloxacin	Temperature-responsive	Silymarine, levofloxacin	Third-degree burn wound	Outstanding shape retention, flexibility abilities, self-healing, wound closure acceleration, inflammation reduction	[153]
Hydrogel based on poloxamers with bio-AgNPs	Temperature-responsive	Bio silver nanoparticle	Chronic infection	Mucoadhesive strength, silver ions release above 37 °C, efficient against Gram-negative, Gram-positive, and fungi	[154]
tea polyphenol silver nanoparticles/phenylboronic acid grafted onto hyaluronic acid–glycol chitosan hydrogels	Glucose-responsive	Tea polyphenol silver nanoparticles	Bacterially diabetic wound	Biological safety, good biocompatibility, shape adaptability, highly efficient antibacterial, anti-inflammatory, collagen deposition and vascularization promotion, and removability	[155]
Metal–organic drug-loaded hydrogel	Glucose-responsive	Zinc ions and deferoxamine mesylate	Diabetic ulcers	Easily applied on the wound surface, zinc ions and deferoxamine mesylate were realized at excessive glucose	[156]
Supramolecular peptide (Nap-Gly-Phe-Phe-Phe-Gly-Val-Asp-CONH_2_) hydrogel doped with nanoparticles	Enzyme-responsive	small interfering RNA-loaded NPs	Diabetic wounds	Improve diabetic healing, regulation of diabetic wound-associated genes	[157]

### 4.2. Biofilm Management

Biofilms are complex accumulations of microbial cells that cultivate on different types of surfaces and are gripped together by an ECM self-generated by the cells. The properties of these biofilms, including their development and morphology, stress and antibiotic resistance, mechanical and chemical attributes, and their effects on the environment and human health, arise from the collective behaviors of the individual cells, each of which can survive and thrive independently. Biofilms represent a major challenge in therapeutic chronic wound infection because they are less vulnerable to antibiotics and can withstand human immunological responses [158,159,160,161].

Instead of focusing on developing new antibiotics or modifying existing ones, the researchers started to explore natural self-defense mechanisms that use enzymes to disturb the integrity of biofilm or induce irreversible damage to bacteria. For example, α-amylase can discompose the biofilm structure by polysaccharide deterioration. Dispersin B and DNase I also represent another promising biofilm matrix degradation, which increases antibiotic penetration and their efficacity on *S. aureus*, *P. aeruginosa*, and *Vibrio chelerae* [77,162,163].

It is already known that the antimicrobial activity of metal nanoparticles (e.g., Ag, CuO, Au, ZnO, zirconium, titanium dioxide) is utilized as single antimicrobial agents in different hydrogel or foam form designs. A combination of nanoparticles and antibiotics can efficiently bust up antibiotic-tolerant biofilm with a minimal dosage of antibiotics. Aspartic acid-ciprofloxacin-polyethylene glycol-coated copper oxide-nanotherapeutics, in an in vivo animal study, eradicated *Staphylococcus aureus* from infected skin. Furthermore, this system presents wound-healing properties [164,165,166].

By combining zinc oxide (ZnO) and titanium dioxide (TiO_2_) with antibiotic amoxicillin-clavulanate, both frameworks displayed greater antibiofilm properties and bactericidal effect on *E. coli 27G* and *A. baumannii 80*, underscoring the promise of utilizing nanoantibiotics in bolstering antibiotic activity against resistant bacteria [167].

Rubio-Canalejas et al. [168] obtained excellent results of a clinical treatment of chronic wound prototype, designed by combining enzymes (DNase I), silver nanoparticles (AgNP), and antibiotics (ciprofloxacin and gentamycin). This approach showed excellent progress in the inhibition of Gram-positive bacteria, *Staphylococcus aureus*, and on Gram-negative bacteria, specifically *Pseudomonas aeruginosa* biofilm formation, in comparison to the use of ciprofloxacin and gentamycin alone. Also, another combination of enzyme-nanoparticles, such as AgNP-α-amylase and AgNP-cellulase, has been studied. However, this combination showed a positive effect on biofilm formation, but was not as effective as AgNP-DNase I in inhibiting biofilm inhibition [168].

### 4.3. Broader Applications

Because of bacterial infections, wound healing can be delayed. Surgical sites can become infected because of several factors, such as biofilm formation, long operative times, emergency cases, or increased blood loss. Due to antimicrobial dressing, the wound can be protected from various bacteria, and the healing process can be quick and effective. Table 8 below represents the different applications of antimicrobial wound application for different surgical site infections [169,170].

The trials assess a variety of therapies, such as regular wound dressings, improved antimicrobial dressings, and various prophylactic approaches that combine medications and dressings. This emphasizes continuous research into tools and methods to enhance surgical results and reduce the risk of infection. Also, the large number of enrolled patients is very significant, meaning the findings are generalizable. The studies in Phase 3 or Phase 4 indicate that the proposed wound care solutions are closer to being used in broader populations.

Table 9 exposes several antimicrobial dressings that are found in the marketplace.

### 4.4. Concerns and Solutions

Even though the utilization of nanoparticles with application in various forms of wound dressing has shown incredible antimicrobial results in infected wounds, there are some concerns regarding the detrimental effects on beneficial bacteria in humans and systemic and local toxic issues [178].

Regarding the cytotoxicity of copper oxide, adding folic acid was proven to slow the release of copper ions, thereby decreasing cytotoxicity and promoting cell migration. It also inhibits pathogenic bacteria like *Klebsiella pneumoniae*, *E. coli*, *Shigella dysenteriae*, *Salmonella typhimurium*, and *S. aureus* [179].

Silver nanoparticles are known for their excellent antibacterial properties against various bacteria ranges, fungi, yeast, and viruses. Also, these nanoparticles tend to aggregate, and they must be immobilized on appropriate support to stop unwanted aggregation. Regarding this, Dong et al. [180] incorporated AgNPs into dialdehyde cellulose nanocrystals to obtain an antimicrobial wound dressing with mechanical strength and a low toxic profile. The results prove that the material seems to be a promising approach for improved antibacterial wound dressing with a low toxic profile and excellent mechanical strength.

Another promising strategy is the utilization of antimicrobial peptides. Antimicrobial peptides (AMPs) have the potential to serve as effective alternatives to traditional antibiotics in the fight against multidrug resistance. Unfortunately, AMPs exhibit toxicity, low selectivity, and limited bioavailability. Regarding this, chitosan is a viable option for AMP encapsulation due to degradation protection during administration and maintain their release. The biocompatible synthetic polymer Poly(lactic-co-glycolic) acid (PLGA) is another polymer that is frequently utilized for AMP encapsulation. Controlled AMP release, cytotoxicity reduction, protection against premature degradation, surface functionalization to concentrate on the infection site, and co-delivery with additional bioactive molecules are some characteristics that PLGA nanoparticles offer for peptide delivery [181,182,183,184].

## 5. Recent Trends in Interdisciplinary Approaches

Nanobiosensors have been developed to monitor wound conditions, such as inflammation or infection signs, with good sensibility and great precision. Smart sensing dressing has gained interest in research due to its capacity to monitor biochemical markers, vital signs, and environmental parameters, enhance real-time, non-invasive evaluation of wound status, and reduce the risk of wound infection. Also, these dressings improve compliance and conformability, while reducing emotional, social, and psychological distress for patients [185,186,187].

Shape memory polymers are a category of porous smart materials that can be deformed and retained in a temporary configuration. They can revert to their original shape when exposed to a specific external stimulus. Polyurethane shape memory polymers (SMP) foams rebound and expand to their first shape to cover the wound and promote clotting when they are applied to the lesion and exposed to the water in the human blood [188,189,190].

Nature is an abundant reservoir of bioactive compounds with significant pharmaceutical properties. Their efficacy and low-toxicity profiles make them gain interest. Also, their natural source typically coincides with conventional medicine procedures, confirming age-old knowledge with contemporary scientific evidence [191,192].

Marine polysaccharides are regarded as excellent materials for wound dressings due to their affordability and widespread availability. Some of the already-known materials are chitosan, alginate, dextran, and hyaluronic acid, which are utilized in various applications in wound dressing [193]. Other examples are salmon roe [194], microalgae [195], and sulfated polysaccharides [196].

Another promising class of materials that has generated significant research interest is silk compound, a promising biomaterial. Silk-like materials, derived from recombinant silk proteins, effectively benefit the natural material and offer enhanced properties, such as scalability, biofunctionalization, tunability, reduced immune response, and improved biocompatibility [197,198,199].

Good biocompatibility, suitable degradation, appropriate mechanical properties, and robust bioactivity are essential characteristics of an ideal scaffold, such as the decellularized extracellular matrix (dECM). dECM is a 3D natural scaffold derived from allogenic, xenogenic, or autologous tissues, where the cellular components were removed without changing their intrinsic tissue structure. dECM, as bioactive hydrogels, could maintain their structural features and stimulatory qualities while simultaneously promoting the seed cells’ migration, growth, proliferation, differentiation, and angiogenesis [200,201].

The future of wound care lies in the synergy of multiple disciplines, combining advanced materials, real-time monitoring technologies, and biological insights to develop smarter and more effective solutions. In addition to improving clinical results, these interdisciplinary approaches concentrate on improving patients’ overall experiences by attending to their emotional and psychological needs and promoting efficient healing processes. We may expect increasingly advanced and sophisticated wound care techniques that take advantage of the greatest aspects of nature and technology, as long as the scientific community keeps developing new ideas.

## 6. Comparative Analysis of Recent Advances

### Hydrogels vs. Foams vs. Antimicrobial Dressings

Given that the wound healing materials outlined above possess distinct structures and diverse applications, Table 10 was created to provide a clear and organized comparison that facilitates a deeper understanding of the differences among each type of wound dressing and specific characteristics.

Table 10 comprehensively illustrates the specific characteristics of hydrogels, foam dressings, and antimicrobial dressings. Each of these three categories of wound care products exhibits unique properties that can significantly affect their efficacy in managing different types of wounds. The selection of an appropriate dressing type depends on various factors, including the wound’s classification—whether it is chronic, acute, infected, or exuding—and the volume of wound exudate, which can be classified as dry, moderate, or high.

Hydrogels are beneficial to dry wounds, while foams are suitable for wounds with moderate to high exudate. Antimicrobial dressings are specifically only for infected wounds. Also, the infection of the wound is an important key factor in choosing the right wound dressing. Because all three categories have the potential to be used as infected wounds, it is necessary to have information about the specific pathogens and their resistance profiles. Also, it is important to know if the patient has any allergies or sensitivities to specific dressing materials. In the end, it is crucial to consider the patient’s comfort; while some patients want to use a foam that can be worn for seven days because they cannot change it daily, some may choose a stimuli-responsive material that can release antimicrobial agents at various biomarkers. By understanding these dressings’ distinct features and functionalities, healthcare providers can make informed choices to optimize healing outcomes for their patients. This tailored approach not only improves patient care but also enhances the overall effectiveness of wound management strategies.

**Figure 5:**
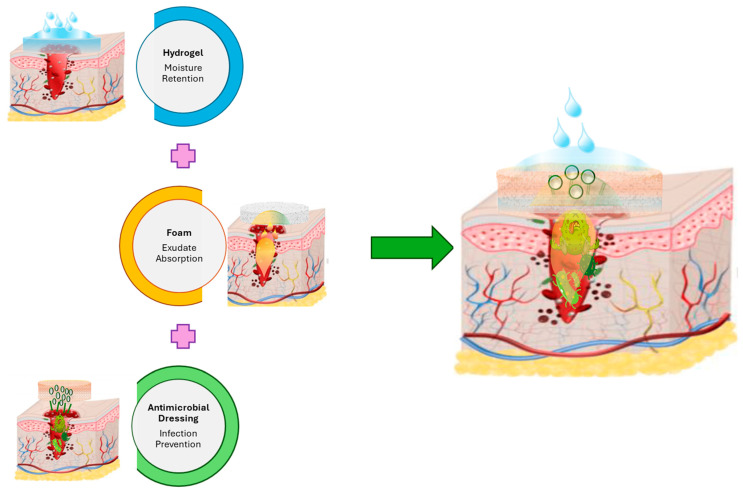
A schematic representation of an antimicrobial hydrogel-foam composite. Created based on information from [26,125,213,214].

The combination of foam, antimicrobial dressings, and hydrogels into new hybrid materials represents a significant advancement in wound care technology, leveraging the unique properties of each component to enhance healing outcomes. By integrating the high moisture-retentive capacity of hydrogels [213] with the effective absorbency of foam dressings [125], the resulting hybrid offers an optimal environment for wound healing. Additionally, incorporating antimicrobial agents into the matrix of such hybrid materials can prevent infection and biofilm production in a wound environment [26]. Figure 5 is a schematic illustration of a hybrid wound dressing that combines the above-mentioned properties.

Regarding the illustration above, some studies have been performed to obtain various hybrid systems by different combinations of hydrogels, foams, and antimicrobial dressings. Table 11 summarizes the reported hybrid systems, their components, and the benefits and applications identified in recent studies.

From Table 11, it can be observed that there is a large interest in obtaining hybrid systems regarding antimicrobial and hybrid/foam dressing, compared to foam and hydrogel types. Also, it can be observed that there is versatility based on various materials that have been utilized. This diversity allows for tailored properties to meet specific clinical needs, whether targeting bacterial infections, promoting cell migration, or controlling moisture levels. In addition, these hybrid systems have been shown to have broad-spectrum antibacterial qualities, improved mechanical and thermal properties, antimicrobial efficacy, anti-inflammatory effects, and better exudate management. These elements are essential to creating efficient wound dressings that support healing while resisting infection.

## 7. Future Directions

### 7.1. Challenges

The development of hybrid materials, regarding the combination of benefits for each type of wound dressing, also has its challenges besides their advancement. One significant barrier is overcoming the cost and the large-scale production that comes with synthesizing this complex hydrogel antimicrobial dressing. Also, regarding the smart wound dressings, besides production scalability, other challenges still exist, such as additional sensor incorporation (biomarkers, pH, metabolites) and clinical translatability (biofouling issues, biocompatibility, for example). There are also obstacles regarding privacy issues, absence of trust, and moral difficulties of patients who utilize one of these devices [221,222,223,224].

Another critical challenge is bridging the gap between laboratory success and clinical adoption. Regarding all the examples above, it can be observed that only a few examples of hydrogel, foam, or antimicrobial dressing are in the advanced stages of clinical trials. Also, many innovative wound care products have been developed, but most are still in the laboratory stage. Factors such as wound types, human social environment, and the need for clinical trials to present their efficacity and safety represent the challenges that need to be resolved to use this wound dressing as a day-by-day medical care [28,225,226]. Despite the multitude of advanced materials, cost, which another important challenge to overcome, comes with integrating various techniques, such as nanomaterials, stimuli-responsive, etc. These innovative approaches inevitably lead to higher production costs, which serve as a significant barrier to their widespread adoption in clinical settings. In addition, regarding the stimuli-responsive antimicrobial dressing, an essential problem is the toxic properties of the materials, particularly inorganic ones, which must be studied and tailored appropriately to make them suitable for use as a clinical product [205,227,228].

### 7.2. Opportunities

Besides these challenges, there are also several opportunities for advancing wound care through innovative dressing technologies. Cutting-edge technologies, including artificial intelligence (AI) and machine learning, have transformed wound care, offering more direct and efficient solutions for both acute and chronic injuries. These technologies can provide early detection, risk stratification, risk factor analysis, prediction, intelligent treatment, diagnosis, and prognosis evaluation, transforming wound care from the study of prevention clinical outcomes in treatments that improve patient care [229,230].

Apart from providing information on wound healing through various applications designed for the real-time home monitoring, artificial intelligence may also have the potential to aid in limb salvage or prevent amputation in cases of lower extremity wounds. By implementing AI in information management, it has significantly changed the education and training of healthcare professionals, providing a variety of advantages, such as chatbots, robots, intelligent tutoring systems, and automated assessment tools. The key feature of AI in medical education is its adaptive learning capability, which analyzes performance and provides instant feedback, helping students identify gaps and receive timely guidance [229,230,231,232].

For example, Swerdlow et al. [233] studied a deep-learning algorithm to segment and classify pressure injury stages. Furthermore, they wanted to verify whether this method has more accurate results than the current one. This method achieves more than 90% accuracy in detection and classification. Chan et al. [234] developed a mobile application using AI-enabled wound imaging to measure the diabetes mellitus wound and compared the results with the traditional method. Their study has demonstrated excellent intra-rater and inter-rater reliability compared to traditional methods. Jiang et al. [224] obtained a miniaturized flexible printed circuit board, a system that includes a lower impedance and adhesive hydrogel electrode. Their study was based on preclinical animal models, and their smart bandage monitored the skin’s physiological signals and provided direct electrical cues, resulting in enhanced dermal recovery, quicker wound closure, and greater neovascularization.

Moreover, expanding personalized wound care with tailor-made dressings based on patient-specific needs is also an opportunity for wound care. Multi-layer dressing, one of the most important advancements, uses various materials to provide a range of advantages, including improved breathability, moisture retention, and higher structural support. Biomaterial dressings have great potential in tissue engineering for aiding tissue repair and regeneration because they can convey signaling molecules, improve skin regeneration, and control the immune response [235].

## 8. Conclusions

In recent years, wound care has gained much interest in obtaining advanced materials, such as hydrogels, foams, and antimicrobial dressing, that can promote wound healing, reduce infection risk, and offer comfort and trust to patients. Additionally, the integration of stimuli-response and artificial intelligence holds great promise for preventing wound infection by delivering antimicrobial agents when there is a deviation from normal conditions and various stimuli.

Despite these promising developments, one of the main challenges remains the gap between laboratory success and clinical utilization. It is necessary to consider the human social environment, the wound type, the patient population, and various clinical trials to obtain clinical validation that can be used for patients in need. Besides innovative and advanced materials, further in-depth studies are required to optimize existing solutions and obtain an ideal wound dressing. Incorporating artificial intelligence, machine learning, and stimuli-responsive technology represents the beginning of customized wound care technologies. Future research may focus on developing dressing designs tailored to each patient’s needs and creating personalized wound dressings that maximize healing efficacy. Another critical aspect that should be taken care of is the cost of production to obtain accessible and reproducible dressing technologies.

To conclude, wound care management benefits from effervescent research interest, giving rise to various hydrogels, foams, antimicrobial dressings, and hybrid materials of great value for healing a wide range of wounds. Despite their scalability and clinical adoption challenges, these materials represent a transformative shift toward patient-centric wound management by integrating comfort, efficacy, and innovation.

## Figures and Tables

**Table 1:** Clinical trials concerning the application of hydrogel-based treatments for chronic wounds, retrieved from ClinicalTrials.gov as of December 2024.

Clinical Trial ID	Official Title	Intervention/Treatment	Enrollment	Study Completion	Phase
NCT02361931	Prospective, Multicenter, Single-blind, Randomized, Controlled Clinical Trial on Safety and Efficacy of a Novel Topical Formulation Containing Erythropoietin for the Treatment of Diabetic Foot Ulcers	Drug: A hydrogel containing erythropoietin Drug: Hydrogel (as a part of SOC)	20	12.06.2018	Phase 1 Phase 2
NCT05607979	The RENEW Study: (Restoring Tissue and Evaluating Novel Treatments for Efficacy in Wounds): A Non-Inferiority Study	Drug: Lavior Diabetic Wound Gel Drug: Smith & Nephew Solosite Gel Hydrogel Wound Dressing	75	01.05.2024	Phase 2 Phase 3
NCT04834245	Evaluation of Diabetic Foot Wound Healing Using Hydrogel/Nano Silver-based Dressing vs. Traditional Dressing: A Prospective Randomized Control Stud	Procedure: Hydrogel/nano silver-based dressing	30	30.12.2019	Not applicable
NCT01143727	Comparison of SANTYL vs. Hydrogel in Debridement of Inflamed Diabetic Foot Ulcers	Drug: Santyl Drug: Tegaderm Hydrogel	20	10.2020	Phase 4
NCT03700580	Application of a Double Blind Clinical Trial Protocol for Evaluation of Healing Action of P1G10, From V Cundinamarcensis to Chronic Neuropathic Wounds in Diabetic Foot Ulcers.	Drug: Hydrogel treatment Drug: P1G10	50	15.10.2016	Phase 2
NCT01427569	A Randomized, Placebo-controlled, Double-blind Phase II Study to Evaluate the Efficacy of IZN-6D4 Gel for the Treatment of Diabetic Foot Ulcers	Drug: IZN-6D4 Gel Other: Placebo hydrogel	82	08.2015	Phase 2
NCT05661474	The Effects of Fitostimoline^®^ Hydrogel Versus Saline Gauze Dressing in Patients With Diabetic Foot Ulcers: a Monocentric, Two-arm, Open-label, Randomized, Controlled Trial.	Drug: Fitostimoline ^®^ hydrogel group Drug: Saline gauze group	40	12.12.2022	Phase 4
NCT03816618	The Healing Effects Of Honey and Hydrogel Products On The Diabetic Foot	Drug: Medihoney Gel in A Tube Drug: Hydrogel Drug: Fucidin Ointment	120	13.02.2023	Early Phase 1
NCT02111291	Clinical Outcomes Associated With Enzymatic Debridement of Diabetic Foot Ulcers for Up To 12 Weeks With Clostridial Collagenase (Santyl^®^) Ointment	Biological: Collagenase SANTYL^®^ Ointment Biological: Hydrogel (if needed) and foam dressing	215	12.2015	Phase 4

**Table 2:** Clinical trials concerning the application of hydrogel-based treatments for burns, retrieved from ClinicalTrials.gov as of December 2024.

Clinical Trial ID	Official Title	Intervention/Treatment	Enrollment	Study Completion	Phase	Location
NCT05877638	Randomized Controlled Trial Assessing a Novel Glycopolymer Compound in the Treatment of Superficial Partial-Thickness Burns	Device: SynePure Wound Cleanser and Catasyn Advanced Technology Hydrogel Drug: SILVADENE Cream 1% (silver sulfadiazine)	2	20.06.2024	Not applicable	Louisiana, USA
NCT03190655	Efficacy and Safety of Aluminaid Versus Hydrogel Wound Dressings in the Treatment of Partial Thickness Burns	Device: Aluminaid Device: Hydrogel	6	05.03.2018	Not applicable	Jakarta, Indonesia
NCT01062191	Flexible Hydrogel Nanoparticle Wound Dressing Allows Greater Joint Range of Motion Compared to Typical Sodium Carboxymethylcellulose Dressing	Device: Aquacel AG, typical carboxymethylcellulose dressing Device: Altrazeal Flexible Hydrogel Nanoparticle Wound Dressing	3	10.2008	Not applicable	Texas, USA
NCT03674151	Randomized-controlled Trial of Wound Healing, Pain, Microbiology, Handling and Thrift of Different Wound Dressings in Patients With Split-skin Grafted Third Degree Burns	Device: Silver Nylon dressing Device: Manuka-Honey Device: Povidone-Iod (PVP-Iod) Device: Hydrogel	20	12.2025 (estimated)	Not applicable	Schleswig-Holstein, Germany
NCT02394873	A Phase 1 Clinical Study to Evaluate the Safety of Allogeneic Adipose-derived Stem Cells in the Subjects With Deep Second-degree Burn Wound	Biological: ALLO-ASC-DFU	5	10.2015	Phase 1	Seoul, South Korea
NCT01499264	Efficacy of MySkin Patch for the Healing of Burn Wounds: a Randomised Controlled Trial	Device: MySkin patch Device: Traditional Dressing	120	20.2014	Phase 3	Milano and Varese, Italy
NCT03183622	A Follow-up Study to Evaluate the Safety for the Patients With ALLO-ASC-DFU Treatment in Phase 1 Clinical Trial of ALLO-ASC-BI-101	Biological: ALLO-ASC-DFU	5	12.2017	-	Seoul, South Korea
NCT04601532	Randomized Controlled Trial Assessing a Novel Glycopolymer Compound in the Treatment of Superficial Partial-thickness Burns	Device: Catasyn™ Advanced Technology Hydrogel and SynePure™ Wound Cleanser Drug: Silver Sulfadiazine	26	08.12.2022	Phase 4	Pennsylvnia, USA
NCT03113747	Safety and Efficacy Evaluation of Tissue Engineered Construct Based on Allogeneic Adipose-derived Multipotent Mesenchymal Stromal Cells and Platelet-poor Plasma Fibrin Hydrogel to Treat the Patients With Burn Wounds	Biological: ALLO-ASCs	20	26.12.2018	Phase 1 Phase 2	Kyiv, Ukraine
NCT03183648	A Follow-up Study to Evaluate the Safety for the Patients With ALLO-ASC-DFU Treatment in Phase 2 Clinical Trial of ALLO-ASC-BI-201	Biological: ALLO-ASC-DFU	14	06.2023	-	Seoul, South Korea

**Table 3:** Examples of hydrogels available on the market.

Commercial Product	Producer	Dressing Type	Type of Wound	Reference
Activheal Hydrogel	Advanced Medical Solutions Ltd.	Amorphous hydrogel	Pressure ulcers, Leg ulcers, Diabetic ulcers, Cavity wounds, Graft and Donor sites, Lacerations and abrasions, Post op surgical wounds	[94]
3M™ Tegaderm™ Hydrogel	Solventum Corporation	Amorphous hydrogel	Pressure ulcers, Arterial ulcers, Venous ulcers, Neuropathic ulcers, Post-operative surgical wounds, Abrasions, Lacerations	[95]
Flaminal	Flen Health	Antimicrobial hydrogel	Superficial and deep partial burns, Infected wounds, diabetic foot ulcers, Leg ulcers, Pressure ulcers, Neonates and Pediatrics, Oncologic wounds, Wounds from Radiotherapy, Skin tears, Post-surgical wounds, Traumatic wounds	[96]
Hydrosorb gel	PAUL HARTMANN AG	Amorphous hydrogel	Ulcers, Decubitus ulcers	[97]
Fitostimoline^®^ idrogel	Damor	Antimicrobial hydrogel	Ulcers, Wounds, Sores, Scalds, Abrasions, First- degree burns, Second-degree burns	[98]
HydroTac^®^	PAUL HARTMANN AG	Sheet of hydrogel	Leg ulcers, Diabetic foot syndrome, Decubital ulcer, Burns up to grade 2A, Skin graft donor sites	[99]
INTRASITE GEL	Smith & Nephew Medical Ltd.	Antimicrobial hydrogel	Pressure sores, Leg ulcers, Diabetic foot ulcers, Malignant wounds, Burns, Surgical wounds, Scalds, Lacerations, Grazes, Amputations	[100]
Iodozyme	Crawford Healthcare Ltd.	Two-layer hydrogel	External wounds, Moderately exuding, Dry wounds, Non-exuding, Under compression therapy, Infected wounds	[101]
Oxyzyme	Crawford Healthcare Ltd.	Two-layer hydrogel	External wounds, Moderately exuding, Dry wounds, Non-exuding, Under compression therapy, Mildly or non-infected wounds	[101]

**Table 4:** Clinical trials concerning the utilization of various foam dressings for venous leg ulcers and infected pressure ulcers, retrieved from ClinicalTrials.gov as of December 2024.

Clinical Trial ID	Official Title	Intervention/Treatment	Enrollment	Study Completion	Phase	Location
NCT01036438	A Double-blind, Comparative, Superiority, Multi-centre Investigation Evaluating the Efficacy of an Absorbent Foam Dressing Containing Silver (Mepilex Ag) Versus the Same Dressing Without Silver Used on Subjects With Venous Leg Ulcers or Mixed Ulcers	Device: Mepilex Ag Device: Mepilex without Ag	201	10.2013	Phase 4	44 locations from (Czech Republic, France, Germany, Netherlands)
NCT00627094	A Randomised, Controlled and Double-blind Clinical Investigation on the Effectiveness and Safety of a Foam Dressing Biatain Ibu Non-adhesive vs. Biatain Non-adhesive, in Painful Chronic Venous Leg Ulcers	Device: Biatain Device: Biatain Ibu	120	04.2009	Not applicable	13 locations from (Denmark, France, Germany, Spain)
NCT02167815	A Multicenter, Post Marketing Clinical Follow up (PMCF) Investigation to Evaluate the Performance and Safety of a Soft Silicone Foam Dressing and to Evaluate the Performance of Standard Care in Exuding Venous Leg Ulcers	Device: Mepilex XT Other: standard care	30	07.2015	Not applicable	3 locations from Prague, Třinec, Jihlava, Czech Republic
NCT05608317	A Prospective, Open, Multi-Center, Interventional, Non-Comparative Clinical Investigation to Follow the Progress of Exuding Venous Leg Ulcers Using a Non-Bordered Foam Dressing	Device: ALLEVYN Non-Adhesive	20	30.11.2024 (estimated)	Not applicable	California, Florida, Pennsylvania, USA
NCT03900455	Effectiveness of the Use of a Polyurethane Foam Multilayer Dressing in the Sacral Area, in Addition to Standard Healthcare, to Prevent the Onset of Pressure Ulcer in Patients at Risk. Multicentric Randomized Controlled Trial	Device: Hydrocellular polyurethane foam multilayer dressing Procedure: Standard preventive care	711	19.03.2020	Not applicable	9 locations from Alessandria, Bologna, Cesena, Pavia, Reggio Emilia, Roma, Trento, Verona, Italy

**Table 5:** Clinical trials concerning the utilization of hydrocellular foam as a treatment for different chronic wounds, retrieved from ClinicalTrials.gov as of December 2024.

Clinical Trial ID	Official Title	Intervention/Treatment	Enrollment	Study Completion	Study Type	Location
NCT03662997	A Prospective, Randomized, Controlled Study Using Cross-Over Design to Evaluate and Compare 3 Multi-Layered Foam Dressings for the Management of Chronic Wounds	Device: Bordered Five-Layer Foam Dressing Device: Hydropolymer Foam Dressing Device: Hydrocellular Multi-Layer Foam Dressing	40	15.11.2019	Interventional	5 locations: California (2), New Jersey (1), Pennsylvania (2), USA
NCT03877484	A Prospective, Multi-center, Post-Market Clinical Follow-Up Study to Evaluate the Safety and Effectiveness of ALLEVYN Gentle Border	Device: ALLEVYN Gentle border	43	19.11.2021	Observational	6 locations: France (1), Germany (4), United Kingdom (1)
NCT03900455	Effectiveness of the Use of a Polyurethane Foam Multilayer Dressing in the Sacral Area, in Addition to Standard Healthcare, to Prevent the Onset of Pressure Ulcer in Patients at Risk. Multicentric Randomized Controlled Trial	Device: Hydrocellular polyurethane foam multilayer dressing Procedure: Standard preventive care	711	19.03.2020	Interventional	9 locations in Italy
NCT03596112	The Difference in Wound Size Reduction Comparing Two Frequently Used Wound Dressings in Everyday Care—a Randomized Controlled Trail	Other: application of a polyacrylate wound pad Other: Hydrocellular foam	77	31.05.2020	Interventional	Onex, Switzerland
NCT02692482	Effectiveness of the Use of a New Polyurethane Foam Multilayer Dressing in the Sacral Area to Prevent the Onset of Pressure Sores in the Elderly With Hip Fractures. Randomized Controlled Trial.	Device: hydrocellular polyurethane foam multilayer dressing Procedure: standard care	359	12.2016	Interventional	Bologna, Italy

**Table 6:** Examples of foam dressings that are commercially available.

Commercial Product	Producer	Dressing Type	Type of Wound	Reference
HydroTac^®^	PAUL HARTMANN AG	PU foam with hydrogel sheet	Small to moderate exudate amount wounds, leg ulcers, diabetic foot syndrome, decubital ulcer, burns up to grade 2A, skin graft donor sites	[99]
3M™ Tegaderm™ Silicone Foam Border	Solventum Corporation	Multi-layer foam dressing	Arterial ulcers, Donor sites, Superficial partial thickness burns, Pressure ulcers, Surgical wounds, Venous leg ulcer	[130]
ALLEVYN	Smith + Nephew	Hydrocellular foam	Burn wounds, Surgical wounds, Skin tears, Donor sites, Diabetic foot ulcers, Venous leg ulcers, Pressure injuries	[131]
ActivHeal^®^ Foam Adhesive	ActivHeal	Two-layer foam dressing	Pressure ulcers, Leg ulcers, Diabetic ulcers, Post-operative surgical, Cavity wounds (secondary dressing), Lacerations and abrasions, Graft wounds, Donor sites, Superficial, partial thickness burns	[132]
Sorbact^®^ Foam Dressing	Abigo Medical AB	bacteria- and fungi foam dressing	Clean, contaminated, colonized, or infected wounds, Surgical wounds, traumatic wounds, Pressure ulcers, Diabetic ulcers, Foot ulcers, Leg ulcers	[133]
PermaFoam^®^ Classic	PAUL HARTMANN AG	Hydrophilic polymer foam dressing	Venous ulcers, Pressure ulcers II-IV, Diabetic foot ulcers, Abrasions, Incisions, Donor sites	[134]
Mepilex^®^ Border Ag	Mölnlycke Health Care AB	Antimicrobial foam dressing	Pressure ulcers, Leg ulcers, Foot ulcers, Partial thickness burns, Traumatic wounds, Surgical wounds	[135]

**Table 8:** Clinical trials concerning different antimicrobial dressings used in surgical site infections and in wounds with multi-drug-resistant pathogens, retrieved from ClinicalTrials.gov as of December 2024.

Clinical Trial ID	Official Title	Intervention/Treatment	Enrollment	Study Completion	Phase
NCT00981110	Surgical Sites Infections Following Colorectal Cancer Surgery. A Randomized Prospective Trial Comparing Standard and Advanced Antimicrobial Dressing Containing Ionic Silver.	Device: AQUAGEL Ag Hydrofiber Wound Dressing Device: Mepore Self-adhesive absorbent dressing	120	12.2010	Phase 3
NCT00203541	Effects of a New Antimicrobial Dressing on Wound Healing and Incidence of Sternal Wound Infections in Subjects Who Have Undergone Cardiac Surgical Procedures Requiring Median Sternotomy	Device: TELFA™ A.M.D. Island dressing	1100	06.2006	Not applicable
NCT03402945	A Cluster-randomized Factorial Crossover Trial, Comparing Antibiotic Mono-prophylaxis With Cefazolin vs. Dual-prophylaxis With Cefazolin Plus Vancomycin and Conventional Wound Dressing vs. Prevena Negative-pressure Wound Management	Device: Prevena Drug: Cefazolin Drug: Vancomycin Other: standard wound dressing	4107	12.2024 (estimated)	Phase 4
NCT03284749	Randomised Controlled Trial on the Effect of Copper Impregnated Dressings and Maternity Pads on the Healing of Obstetric Wounds and Wound Infection	Other: Copper impregnated wound dressing Other: Normal wound dressing Other: Copper impregnated maternity pads Other: Normal maternity pads	774	19.12.2017	Not applicable

**Table 9:** Examples of commercially accessible antimicrobial dressings.

Commercial Product	Producer	Dressing Type	Type of Wound	Reference
Atrauman Ag	Paul Hartmann AG	Silver-containing dressing	Ulcus cruris, Diabetic leg ulcer, Decubitus, Acute burns (up to 2nd degree)	[171]
Suprasorb A + Ag	Lohmann & Rauscher	Silver-containing dressing	Venous leg ulcers, Arterial ulcers, Diabetic ulcers, Pressure ulcers	[172]
Aquacel AG+ Extra	ConvaTec	Silver-containing dressing	Diabetic foot ulcers, Leg ulcers, Venous stasis ulcers, Arterial ulcers, Leg ulcers of mixed etiology, Pressure ulcers/sores, Surgical wounds, Traumatic wounds, Fungoides-cutaneous tumors, Fungating carcinoma, Kaposi’s sarcoma, Angiosarcoma, Cutaneous metastasis	[173]
Telfa	Cardinal Health	Non-adherent antimicrobial dressings	Lightly draining wounds, Burns, Skin grafts, Donor sites, Abrasions, Surgical incisions, Chronic wounds	[174]
3M™ Silvercel™	3M	Silver-containing dressing	Chronic wounds with moderate to high levels of exudate	[175]
Askina^®^ Calgitrol^®^ Ag+	B. Braun SE	Sil-ver-containing dressing	Leg ulcers, Pressure ulcers, Diabetic ulcers, Post-operative, Wounds left to heal by secondary intent, Donor sites, Abrasions, Lacerations, Partial thickness burns	[176]
Cutimed^®^ Sorbion^®^ Sorbact^®^	Abigo Medical AB	Antimicrobial dressings	Pressure ulcers, Leg ulcers, Diabetic foot ulcers, Surgical wounds, Traumatic wounds	[177]

**Table 10:** Comparison of hydrogels, foams, and antimicrobial dressings.

Type of Dressings	Composition	Patient Comfort	Properties	Recent Innovation	Targeted Wounds	References
Hydrogels	Natural, synthetic polymers	Easy dressing removal	Absorb excess exudate, good biological compatibility, good hemostatic, tissue repair stimulation, gas exchange, strongly adhere to	Bioactive agents’ incorporation	Acute and chronic wounds	[36,74,202,203,204,205]
Foam Dressings	Polyurethane, silicone resins	Depending on the exudate amount, it can be worn for one to seven days with silicone adhesive, easily removed, lower pain risk and skin stripped	Absorb high to moderate wound exudate, maintain moisture, control gaseous exchange, effective thermal insulation, cushioning, protection	Antimicrobial agent incorporation	Acute and chronic wounds	[115,206,207,208,209]
Antimicrobial Dressings	Different antimicrobial agents	Good tolerability	Effective against various types of bacteria, fungi, fungi, viruses, low- to moderate-exuding wounds	Stimuli response smart dressing	Diabetic wound, surgical site infections	[144,210,211,212]

**Table 11:** Examples of various hybrid systems, their components, benefits, and applications.

Hybrid System	Components	Benefits	Reported Efficacy	Application Areas	Reference
Hydrogels and antimicrobial dressing	Hyaluronic acid-methacrylic anhydride, antimicrobial peptides, tetrahedral framework nucleic acid	Antimicrobial activity, anti-inflammatory	Broad-spectrum antibacterial properties, cell migration promotion, anti-inflammatory properties, reduced scarring	Infected wound	[215]
Antimicrobial polyurethane foam	Polyurethane foam, castor oil, zinc oxide	Antibacterial activity	Improvement of thermal and mechanical properties, antimicrobial activity against (*P. aeruginosa*)	Hospital infections	[216]
Temperature-responsive hydrogel	Temperature-responsive hydrogel, oxidized chondroitin sulfate, gelatin, silver nanoparticles	Antimicrobial, temperature-responsive properties, exudate absorption	Efficient exudate absorption, reduction in skin infection, superior adhesion on skin at 37 °C, non-cytotoxic, superior viscoelastic properties	Diabetic wound infections	[217]
Hydrogel + foam	Poly (ethylene glycol) diacrylate based	Wound moisture balance, remove exudate	Self-tuning moisture control, remove exudate fast	Exudative and dry chronic wounds	[218]
Foam-based antibacterial hydrogel	Carboxymethyl cellulose, polyvinyl alcohol, cerium oxide nanoparticle	Antibacterial activity, exudate absorption, biodegradability, and drug delivery	Effective antibacterial properties (*E. coli* and *S. aureus*), good swelling properties (foam gel’s swelling ratio to %1057 in just one hour), drug delivery characteristics (silver sulfadiazine up to 6 h)	Wound infection	[219]
Antimicrobial hydrogel foam dressing	Poly (lactic-co-glycolic acid) (PLGA) microsphere, gallium maltolate	Drug release profile, cytocompatibility, antimicrobial activity	low cytotoxicity on dermal fibroblasts, a consistent release profile throughout the desired five days, gallium maltolate prohibited bacterial growth (both drug-susceptible and resistant)	Chronic wound	[220]

## Data Availability

No new data were created or analyzed in this study.
